# Increased Risk of Incident Uveitis Among Patients with Psoriasis: A Nationwide Population-Based Cohort Study

**DOI:** 10.3390/diagnostics16040627

**Published:** 2026-02-21

**Authors:** Scott Ehrenberg, Yoav Elizur, Niv Ben-Shabat, Paula David, Kassem Sharif, Yossef S. Bernstein, Ibrahim Abu Hilwe, Arnon D. Cohen, Abdulla Watad, Howard Amital, Yonatan Shneor Patt

**Affiliations:** 1 Department of Internal Medicine B, Sheba Medical Center, Tel-Hashomer, Ramat Gan 5262100, Israel; scottehrenberg@gmail.com (S.E.); yoav.elizur@mail.huji.ac.il (Y.E.); nivben7@gmail.com (N.B.-S.); paulardavid@gmail.com (P.D.); abuhilwe2@gmail.com (I.A.H.); watad.abdulla@gmail.com (A.W.); howard.amital@sheba.health.gov.il (H.A.); 2 Gray Faculty of Medical and Health Sciences, Tel-Aviv University, Tel-Aviv 6927846, Israel; kassemsharif@gmail.com; 3 Dina Recanati School of Medicine, Reichman University, Herzliya 4610101, Israel; 4 Section of Musculoskeletal Disease, The National Institute for Health and Care Research (NIHR) Leeds Musculoskeletal Biomedical Research Unit, Chapel Allerton, Leeds Teaching Hospital Trust, Leeds LS1 3EX, UK; 5 Center for Advanced Endoscopy, Beth Israel Deaconess Medical Center and Harvard Medical School, Boston, MA 02108, USA; 6 Faculty of Medicine, Hebrew University of Jerusalem, Jerusalem 91120, Israel; yossef.berenshtain@mail.huji.ac.il; 7 Chief Physician’s Office, Clalit Health Services, Tel-Aviv 6209813, Israel; 8 Siaal Research Center for Family Medicine and Primary Care, Faculty of Health Sciences, Ben-Gurion University of the Negev, Beer-Sheva 8410501, Israel; 9 Rheumatology Unit, Sheba Medical Centre, Tel-Hashomer 5262100, Israel; 10 Education Authority, Sheba Medical Center, Tel-Hashomer 5262100, Israel

**Keywords:** psoriasis, psoriatic arthritis, spondyloarthritis, uveitis, inflammation, epidemiology, biologics

## Abstract

**Background:** Psoriasis is a chronic systemic inflammatory disease with established extra-cutaneous manifestations. While the association between uveitis and spondyloarthritis (SpA)-related disorders is well recognized, the incident risk of uveitis among broader psoriasis populations remains inadequately defined due to methodological limitations and inconsistent findings across previous studies. We aimed to estimate the incidence of uveitis in a large, nationwide population-based cohort and identify specific clinical and treatment-related predictors of ocular inflammation. **Methods:** This retrospective cohort study utilised electronic health records from Clalit Health Services, Israel’s largest health maintenance organization (2002–2024). We identified 157,360 patients with dermatologist-confirmed psoriasis and 156,927 age- and sex-matched controls. The primary outcome was incident uveitis, with risk estimated using Cox proportional hazards models. Within the psoriasis cohort, multivariable logistic regression was employed to identify predictors of uveitis, ensuring appropriate temporal sequencing between psoriasis treatment exposure and outcome. **Results:** Over a median follow-up of 12.6 years, psoriasis was associated with a significantly higher risk of incident uveitis (adjusted Hazard Ratio [aHR] 1.80; 95% CI, 1.50–2.15). Stratified analysis revealed a graded risk pattern: mild psoriasis showed no increased risk (aHR 1.01; 95% CI, 0.91–1.13), whereas severe disease (aHR 1.59; 95% CI, 1.25–2.03) and concomitant SpA (aHR 2.21; 95% CI, 1.87–2.61) demonstrated markedly elevated risks. Within the psoriasis cohort, independent predictors included SpA, diabetes mellitus, systemic lupus erythematosus, and sarcoidosis. Exposure to biologics, particularly etanercept (OR 3.37; 95% CI, 2.42–4.54), was associated with higher odds of uveitis, potentially reflecting higher disease severity. **Conclusions:** Incident uveitis risk in psoriasis is primarily driven by the magnitude of systemic inflammatory burden, with the highest risk observed in severe disease and those with concomitant SpA. Clinicians should maintain heightened vigilance for ocular symptoms in these high-risk subgroups to ensure timely intervention.

## 1. Introduction

Psoriasis is a chronic immune-mediated inflammatory skin disorder affecting approximately 3% of the global population and is associated with multiple comorbid conditions, including psoriatic arthritis (PsA), cardiovascular disease, metabolic syndrome, and inflammatory bowel disease (IBD) [1,2,3]. Reflecting its systemic nature, psoriasis has also been linked to a range of ocular manifestations involving the cornea, conjunctiva, and eyelids which may present as blepharitis, dry eye disease, and corneal scarring [4]. Management is guided by disease severity and patient preferences, encompassing conventional approaches such as topical therapies, phototherapy, and systemic medications, alongside newer biologic agents [5,6].

Uveitis is an inflammatory eye disease characterized by inflammation of the uveal tract, comprising the iris, ciliary body, and choroid. Although uveitis is most often immune-mediated, it may also be infectious or idiopathic [7]. Importantly, uveitis represents a major public health burden and is estimated to account for a meaningful proportion of blindness in developed countries. Delayed diagnosis or insufficient treatment can lead to serious complications and permanent vision loss. Uveitis also disproportionately affects individuals of working age, contributing to substantial functional impairment and socioeconomic consequences [8,9].

The association between uveitis and spondyloarthritis (SpA)-related disorders, including IBD, axial SpA and PsA, is well established; in this context uveitis has been linked to a more severe disease course and higher inflammatory activity [7,8,10,11,12]. In contrast, the relationship between uveitis and psoriasis in the absence of concomitant SpA remains less clearly defined. Existing evidence regarding this association is limited by several methodological gaps. First, because uveitis is a relatively rare disease, low event counts in most cohorts have resulted in limited statistical power, often necessitating reliance on meta-analyses to obtain more precise pooled effects [13,14,15,16]. However, even the few available population-based studies have reported inconsistent subgroup findings. In several reports, an increased risk was observed primarily among patients with severe psoriasis and concomitant SpA, whereas no association was demonstrated in psoriasis without these characteristics [17]. Furthermore, a substantial proportion of the existing literature comprises cross-sectional studies, focusing on prevalence rather than incident risk and thereby limiting inference regarding temporal relationships [11].

Considering these literature gaps and given the clinical value of risk stratification and uveitis prevention, we utilized a large population-based cohort from Clalit Health Services (CHS), Israel’s largest health maintenance organization, to estimate the incidence of uveitis among patients with psoriasis. We further aimed to identify psoriasis subgroups at increased risk and to evaluate the association of demographic factors, comorbid conditions, and treatment exposures with subsequent uveitis.

## 2. Methods

### 2.1. Ethics

This study was approved by the Institutional Ethics Committee of CHS, Tel Aviv, Israel (Approval No. 0212-17-COM; initial approval granted on 3 January 2018, with an extension valid through 15 April 2026). As the research was based on de-identified data extracted from electronic health records, the requirement for informed consent was formally waived by the committee.

### 2.2. Study Design

This was a retrospective cohort study based on electronic health records from CHS, conducted to evaluate the risk of uveitis among patients with psoriasis, including analyses across clinically relevant subgroups.

### 2.3. Settings

This study was conducted using data from CHS, which operates under the Israeli National Health Insurance Law enacted in 1994, ensuring universal health coverage for all residents regardless of age, sex, or health status. Enrolment in one of four national health maintenance organizations (HMOs) is mandatory, with CHS being the largest, serving approximately 4.5 million individuals across diverse ethnic and sociodemographic groups. CHS functions as both insurer and provider and maintains a comprehensive, centralized electronic health record system. This database integrates longitudinal medical data from community-based and hospital settings, including physician diagnoses, medication dispensations, laboratory results, outpatient visits, emergency department use, and inpatient discharge summaries. The CHS database has been validated and extensively utilized in epidemiological and clinical research, with numerous peer-reviewed publications supporting its utility as a robust real-world data source [18,19].

### 2.4. Population and Study Design

We identified all individuals with a dermatologist-confirmed diagnosis of psoriasis recorded in CHS between1 January 2002, and 31 December 2024. Diagnoses were identified using International Classification of Diseases, Ninth Revision (ICD-9) codes, as implemented within the CHS administrative and research databases during the study period. Although ICD-10 has been globally adopted, the transition from ICD-9 to ICD-10 has occurred at different times across healthcare systems worldwide. Within CHS, ICD-9 coding remained the standard classification framework in administrative databases throughout much of the study period; therefore, case definitions for psoriasis and uveitis were based on ICD-9 codes. Psoriasis cases were defined using ICD-9 codes 696, 696.0, and 696.1. For each case, a control subject without a recorded diagnosis of psoriasis was selected, matched 1:1 by sex and date of birth. Follow-up continued until January 1, 2025. The incidence of uveitis, identified by specialist-assigned ICD-9 codes, was compared between the psoriasis and control groups over the follow-up period.

### 2.5. Variables and Measures

Study variables were obtained from electronic medical records maintained by CHS. Demographic information included age, sex, and ethnicity, which was categorized as Jewish or Arab. The primary outcome was the incidence of uveitis, identified by ICD-9 diagnosis codes (363.* and 364.*). SpA-related comorbidities, including PsA (ICD-9 codes 696.0, 713.3), ankylosing spondylitis (AS, ICD-9 720.), and inflammatory bowel diseases (IBD, Crohn’s disease [ICD-9 555.*] and ulcerative colitis [ICD-9 556.*]), were identified using the same ICD-9 coding framework. Body mass index (BMI) was calculated from the most recent height and weight measurements available prior to or near the psoriasis diagnosis date and was categorized as <30 or ≥30 kg/m^2^.

Chronic comorbid conditions, such as diabetes mellitus (DM), ischemic heart disease (IHD), cerebrovascular accident (CVA), congestive heart failure (CHF), chronic obstructive pulmonary disease (COPD), and malignancy, were ascertained from Clalit’s chronic disease registry and administrative databases. Systemic treatments and phototherapy were extracted from pharmacy dispensing records following the diagnosis of psoriasis and classified as ever vs. never exposed. Treatment categories included phototherapy, cyclosporine, methotrexate, tumor necrosis factor (TNF) inhibitors, IL-12/23 inhibitors, and IL-17 inhibitors. Psoriasis severity was defined according to treatment modality, with patients receiving systemic agents or phototherapy classified as having severe disease, and those treated exclusively with topical therapy considered to have mild disease.

### 2.6. Statistical Analysis

Continuous variables were summarized as mean ± standard deviation and compared using Student’s *t*-test. Categorical variables were presented as percentages and compared using Pearson’s chi-square test.

Two primary analyses were undertaken to assess the relationship between psoriasis and incident uveitis. In the first analysis, we compared the incidence of new-onset uveitis between patients with psoriasis and age- and sex-matched controls. The index date was defined as the date of psoriasis diagnosis. To ensure that all individuals were at risk at cohort entry, participants with a documented diagnosis of uveitis prior to the index date were excluded. Incidence rates were calculated per 1000 person-years. Time-to-event analyses were conducted using Kaplan–Meier-based cumulative incidence curves, and differences between groups were assessed with the log-rank test. Cox proportional hazards regression models were used to estimate hazard ratios (HRs) and corresponding 95% confidence intervals (CIs).

In the secondary analysis, the sample was restricted to patients with psoriasis without pre-existing uveitis at baseline, in order to evaluate potential predictors of uveitis within this population. Univariable and multivariable logistic regression models were employed to examine associations between clinical, demographic, and treatment-related factors and subsequent development of uveitis. For treatment-related variables, individuals whose first recorded exposure to a given therapy occurred after the onset of uveitis were excluded using row-wise exclusion, ensuring appropriate temporal alignment between exposure and outcome.

All statistical tests were two-sided, and a *p* value < 0.05 was considered statistically significant. All analyses were performed using R Statistical Software (version 4.4.2).

## 3. Results

### 3.1. Study Population

A total of 157,360 patients with psoriasis and 156,927 age- and sex-matched controls were included in the study (Table 1). The groups were comparable in age (mean 47.8 vs. 47.7) and had a similar sex distribution (approximately 50% male in both groups). Arab ethnicity was less frequent among patients with psoriasis than among controls (14.9% vs. 19.8%). In addition, obesity (32.8% vs. 27.7%) and current smoking (44.3% vs. 38.7%) were significantly more common among patients with psoriasis compared to controls (both *p* < 0.001).

Psoriasis patients had a higher burden of cardiometabolic comorbidities, including IHD (15.9% vs. 12.4%), CHF (6.8% vs. 4.4%), DM (23.5% vs. 20.2%), hypertension (35.7% vs. 32.4%) and CVA (7.6% vs. 6.0%). Several additional comorbidities were more common in the psoriasis group, including chronic renal failure (CRF) (0.7% vs. 0.5%), malignancy (15.6% vs. 11.9%), COPD (6.1% vs. 3.8%), and asthma (8.7% vs. 6.9%) (all *p* < 0.001).

Immune-mediated inflammatory diseases were also more prevalent among psoriasis patients, including uveitis (1.0% vs. 0.8%), ulcerative colitis (UC) (0.7% vs. 0.5%), Crohn’s disease (CD) (1.0% vs. 0.5%), rheumatoid arthritis (RA) (1.8% vs. 1.0%), systemic lupus erythematosus (SLE) (0.4% vs. 0.2%), scleroderma (0.2% vs. 0.1%) and sarcoidosis (0.3% vs. 0.2%) (all *p* < 0.001).

### 3.2. Characteristics of Uveitis Cases Within the Psoriasis Cohort

Among the 157,360 patients with psoriasis in the cohort, 1512 (1.0%) were diagnosed with uveitis. The mean age at psoriasis diagnosis was 53.2 years (±17.6), and the mean age at uveitis diagnosis was 54.3 years (±17.9); 47.0% were male and 14.7% were of Arab ethnicity. Most uveitis cases occurred in patients with mild psoriasis (81.9%), while 18.1% had severe disease (Table 2).

SpA-related comorbidities were frequent, including inflammatory arthritis (17.9%) and IBD (5.2%). The temporal relationship between psoriasis and uveitis differed substantially across patients. In 44.8% of cases, uveitis preceded the diagnosis of psoriasis, while in 55.2% it was diagnosed subsequently. The median time interval between diagnoses was 5.4 years (range, 0–21 years). Regarding treatment, 8.3% of patients had received phototherapy and 15.7% were treated with methotrexate. Biologic exposure included anti–TNF agents (13.6%), IL-12/23 inhibitors (1.6%), IL-17 inhibitors (2.3%), and IL-23 inhibitors (0.7%); 3.6% had received more than one class of biologic therapy.

### 3.3. Uveitis Risk in Psoriasis and Subgroups

In the time-to-event analysis (156,682 patients with psoriasis and 156,434 matched controls), median follow-up was 12.6 years (IQR, 7.8–17.7) in both groups (Table 3). During follow-up, the incidence of uveitis was higher in patients with psoriasis compared to matched controls. Among 156,682 patients with psoriasis, 834 developed uveitis over 1,990,558 person-years (incidence rate: 0.419 per 1000 person-years), compared to 702 events among 156,434 controls over 1,985,618 person-years (0.354 per 1000 person-years). In unadjusted Cox models, psoriasis was associated with a higher risk of uveitis (HR, 1.19; 95% CI, 1.07–1.31), with a larger estimate after age and sex adjustment (aHR, 1.80; 95% CI, 1.50–2.15).

Risk differed across clinically relevant psoriasis subgroups. Mild psoriasis was not associated with increased uveitis risk compared with controls (incidence rate, 0.361 per 1000 person-years; adjusted HR, 1.01; 95% CI, 0.91–1.13). By contrast, severe psoriasis was associated with higher risk (incidence rate, 0.532 per 1000 person-years; aHR, 1.59; 95% CI, 1.25–2.03), and the highest risk was observed in psoriasis with SpA-related comorbidities (incidence rate, 0.773 per 1000 person-years; adjusted HR, 2.21; 95% CI, 1.87–2.61).

**Figure 1:**
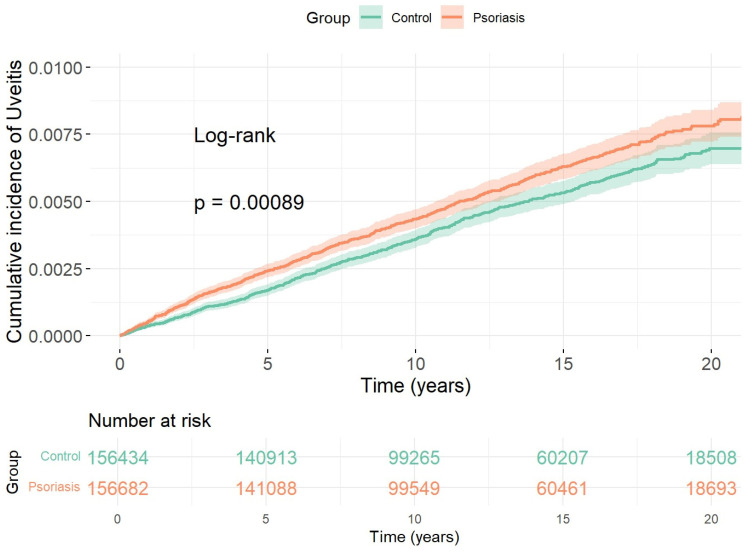
Cumulative incidence of uveitis in psoriasis versus matched controls. Kaplan–Meier curves comparing the cumulative incidence of uveitis among patients with psoriasis (*n* = 156,682) and age- and sex-matched controls (*n* = 156,434) over a median follow-up of 12.6 years. The cumulative incidence was significantly higher in the psoriasis group (adjusted HR 1.80; 95% CI, 1.50–2.15), supporting an elevated risk of uveitis associated with psoriasis.

**Figure 2:**
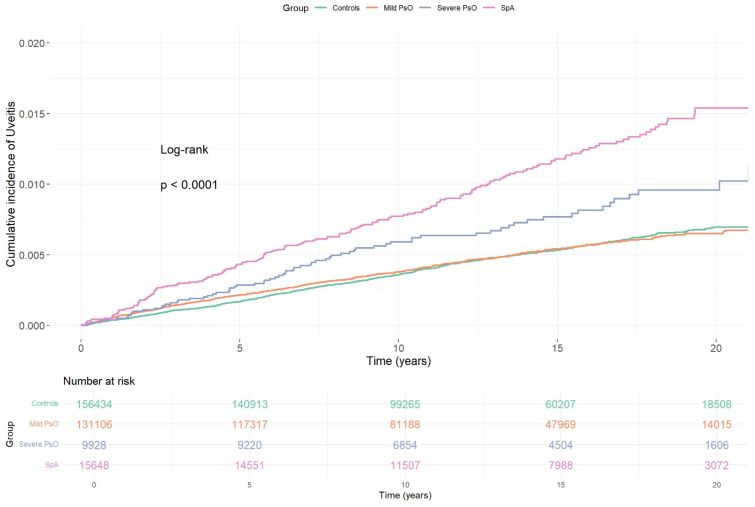
Cumulative incidence of uveitis across psoriasis subgroups versus matched controls. Kaplan–Meier curves showing the cumulative incidence of uveitis stratified by psoriasis severity and SpA-related comorbidities compared to matched controls. While patients with mild psoriasis showed no excess risk (adjusted HR 1.01; 95% CI, 0.91–1.13), those with severe psoriasis (aHR 1.59; 95% CI, 1.25–2.03) and those with SpA-related conditions (aHR 2.21; 95% CI, 1.87–2.61) exhibited significantly increased uveitis risk.

The Kaplan–Meier estimates mirrored the Cox findings. The cumulative incidence curve for the overall psoriasis cohort separated from that of matched controls, while subgroup curves indicated that separation was driven by severe psoriasis and psoriasis with SpA-related comorbidities; in contrast, the mild psoriasis curve largely overlapped the control curve (Figure 1 and Figure 2).

### 3.4. Predictors of Uveitis in the Psoriasis Cohort

In multivariable logistic regression restricted to patients with psoriasis, SpA and several systemic comorbidities emerged as independent predictors of uveitis. Specifically, higher odds of uveitis were observed in patients with SpA (OR, 1.83; 95% CI, 1.40–2.37) and DM (OR, 1.53; 95% CI, 1.22–1.92). In addition, SLE (OR, 2.46; 95% CI, 1.00–5.44) and sarcoidosis (OR, 7.14; 95% CI, 3.84–12.10) were associated with markedly higher odds of uveitis.

In contrast, demographic factors (age and Arab ethnicity) and several cardiometabolic comorbidities (including obesity, hypertension, IHD, CHF, CVA, and malignancy) did not retain independent associations in the multivariable model; female sex showed a numerically higher odds ratio, although this did not reach statistical significance.

Pre-event treatment exposures differed in the magnitude of the association with uveitis. Exposure to cDMARDs was associated with increased odds (OR, 1.77; 95% CI, 1.42–2.17), while monoclonal anti-TNFα exposure showed a higher OR (2.29; 95% CI, 1.72–2.98). The highest odds were observed with etanercept (OR, 3.37; 95% CI, 2.42–4.54). In contrast, phototherapy and anti-IL-17 exposure were not associated with higher odds in the multivariable model (detailed results are presented in Table 4).

## 4. Discussion

In this large, population-based nationwide cohort with a median follow-up of 12.6 years, we observed a significantly higher incidence of uveitis among patients with psoriasis compared to matched controls (1.0% vs. 0.8%, *p* < 0.001; HR 1.80, 95% CI 1.50–2.15). Subgroup analyses revealed that patients with concomitant SpA exhibited the highest risk of developing uveitis (HR 2.21, 95% CI 1.87–2.61), followed by those with severe psoriasis (HR 1.59, 95% CI 1.25–2.03), whereas patients with mild psoriasis demonstrated no increased risk compared to controls (HR 1.01, 95% CI 0.91–1.13). This graded risk pattern suggests that uveitis risk increases in proportion to the severity of the underlying inflammatory condition.

The potential relationship between psoriasis and intraocular inflammation was first proposed in 1979 by Knox, who described a case series of ten patients with both conditions, most of whom did not exhibit clinical arthritis [14]. Since that initial observation, several studies have explored this association; however, most were limited by small sample sizes and cross-sectional or case–control designs, resulting in inconsistent findings. For example, an Egyptian study involving 100 patients with psoriasis and 100 matched healthy controls identified three cases of anterior uveitis within the psoriasis group, all of whom also had SPA, but the difference did not reach statistical significance [20]. In contrast, a larger-scale cross-sectional study including 37,456 individuals with psoriasis reported a significant association between psoriasis and uveitis, with a prevalence ratio of 1.95 (95% CI 1.66–2.29) [21].

Subsequently, only a limited number of large-scale cohort studies have evaluated the incidence of uveitis among patients with psoriasis. A retrospective cohort study from Taiwan involving 147,954 patients with psoriasis demonstrated that higher levels of inflammatory activity were associated with a stepwise increase in the risk of incident uveitis. Increased risk was observed among patients with severe psoriasis without SpA and among those with mild psoriasis with SpA, whereas no association was found in patients with mild psoriasis without SpA [22]. A similar conceptual framework was supported by a nationwide cohort study from Korea involving 317,940 patients with psoriasis, which reported an increased risk of both incident and recurrent uveitis across psoriasis severity strata, with the highest risk observed in patients with concomitant SpA [23]. In contrast, a Danish population-based study reported elevated uveitis risk among patients with mild psoriasis and those with SpA, but notably not among those classified as having severe psoriasis [17]. This finding differs from the previous cohorts, in which uveitis risk increased in parallel with greater inflammatory severity.

Moreover, in a recent retrospective population-based cross-sectional study conducted by our group using data from a smaller HMO in Israel, which included 61,003 patients with psoriasis, an increased prevalence of uveitis was observed only in the presence of SpA-related conditions and not among patients with severe psoriasis alone [11]. In that study, we hypothesized that this discrepancy may be partly attributable to genetic variability within the Israeli population, which is characterized by a lower prevalence of pro--inflammatory genetic markers, and consequently, a lower risk of uveitis [24]. Alternatively, the smaller sample size of our previous cohort may have limited statistical power, suggesting that the observed differences may reflect methodological constraints rather than true biological variation.

Considering these conflicting conclusions, the present study provides a substantial contribution to the current understanding of the relationship between psoriasis and uveitis. By leveraging data from the largest HMO in Israel and employing a longitudinal, incidence-based design with extended follow-up, we were able to quantify risk rather than cross-sectional association. Our findings reinforce the concept that systemic inflammatory burden is the primary driver of uveitis risk among psoriasis patients. Importantly, although this study was conducted within a single national healthcare system, it draws from a population characterized by substantial ethnic and genetic heterogeneity, including Jewish and Arab populations and diverse ancestral origins, which may enhance the generalizability of our results to other multiethnic settings.

Beyond measuring overall risk, we also sought to identify specific predictors of uveitis development within the psoriasis cohort. When conducting multivariable analyses, female sex showed a trend toward a higher odds ratio for uveitis, although the estimate did not reach statistical significance. This finding is consistent with the broader female predominance observed across several immune-mediated diseases and may reflect sex-related differences in immune regulation [25]. Consistent with the immunologic basis of uveitis, immune-mediated comorbidities, including SpA, SLE, and sarcoidosis, were independently associated with uveitis. Furthermore, DM was also identified as an independent predictor, consistent with findings from our previous study [11]. Collectively, these patterns align with existing literature on systemic inflammatory and autoimmune conditions associated with uveitis, thereby supporting the robustness and external validity of our cohort-based estimates, and reinforcing the study’s central hypothesis that elevated inflammatory activity contributes to uveitis development [26,27,28].

In this study, we also investigated the impact of systemic psoriasis therapies on the risk of developing new-onset uveitis, an analysis that, to our knowledge, has not been previously conducted with appropriate temporal alignment between treatment initiation and subsequent uveitis onset. Notably, we observed that exposure to cDMARDs was associated with an increased risk of uveitis, whereas the risk was substantially higher among patients treated with biologic agents, particularly those receiving etanercept.

In our previous study, which examined treatment-uveitis relationships using a cross-sectional design based on prevalence, we observed that exposure to biologic agents was associated with increased odds of uveitis [11]. However, that analysis was inherently limited by its inability to distinguish whether systemic therapies were initiated before or after uveitis onset. Given that TNFα inhibitors are frequently prescribed for psoriasis patients with uveitis, biologic use in that context may have reflected treatment in response to the ocular complication rather than a contributing risk factor [29]. In the current study, we addressed this limitation by restricting the analysis to treatment exposure that occurred prior to the uveitis event, thereby enabling a more accurate estimation of treatment-related risk.

Surprisingly, despite their anti-inflammatory mechanism of action, biologic agents did not appear to mitigate the risk of uveitis in our cohort; instead, they were linked to higher odds of uveitis development. This finding may be explained by confounding related to disease severity. In our cohort, treatment intensity served as a proxy for psoriasis severity due to the absence of direct clinical metrics such as Psoriasis Area and Severity Index (PASI). Thus, patients receiving biologics likely represent a subgroup with more severe systemic inflammation, which may itself be the principal driver of uveitis risk, potentially offsetting any protective effect of treatment. To more accurately assess the impact of monoclonal TNF inhibitors, future studies should compare patients with similar levels of psoriasis severity, ideally measured by PASI scores, who did or did not receive these agents.

Interestingly, a recent study by Hijazi et al. [12] investigated the incidence of uveitis within a PsA cohort. Notably, their multivariable analysis identified etanercept exposure as an independent risk factor for uveitis, whereas monoclonal anti-TNFα agents were not significantly associated with increased risk. The authors interpreted their findings in light of prior evidence, suggesting that etanercept may be less effective in preventing non-infectious uveitis and, in some cases, may even precipitate de novo uveitis in patients with SpA, whereas monoclonal TNF inhibitors may reduce the risk of uveitis development [30,31,32]. However, a closer examination of their results reveals a clear trend toward increased risk in PsA patients treated with monoclonal anti-TNFα agents, with a hazard ratio of 1.53 (95% CI, 0.95–2.47, a borderline *p*-value = 0.077), a finding that did not reach statistical significance, likely due to the limited power of their smaller cohort. By comparison, our study, which included a substantially larger sample size of patients with psoriasis, demonstrated that both etanercept and monoclonal anti-TNFα therapies were independently associated with higher odds of developing uveitis, with the greatest risk observed in patients treated with etanercept. These findings support the hypothesis that etanercept may carry a distinct risk profile in the context of ocular inflammation, and the elevated risk seen with monoclonal TNF inhibitors may serve as a marker of underlying disease severity rather than a direct causal factor.

The association between psoriasis and uveitis may be attributed to overlapping immunopathogenic pathways. Uveitis is characterized by immune-mediated intraocular inflammation involving ocular dendritic cells, IFN-γ-producing Th1 cells, and IL-17-producing Th17 cells, with Th17 activation through STAT3 signaling contributing to disease onset, progression, and disruption of the blood-retinal barrier [33,34,35,36]. Likewise, psoriasis is driven by Th17-mediated keratinocyte hyperproliferation and Th1-dependent activation of dendritic cells, resulting in IL-23-mediated differentiation of naïve CD4^+^ T cells into Th17 cells [37,38,39]. Furthermore, several proinflammatory cytokines implicated in psoriasis, including IL-2, IL-6, and TNF, have been found at elevated levels in the aqueous humor of patients with uveitis, reinforcing the presence of shared inflammatory mechanisms [40,41].

This study has several limitations inherent to its retrospective design and reliance on registry-based data derived from electronic health records using ICD-9 coding. First, the dataset did not include detailed clinical measures of psoriasis severity, such as histopathological characteristics or PASI scores, which are considered standard tools for assessing disease severity. Consequently, psoriasis severity was inferred based on treatment patterns, an approach that may not accurately reflect clinical severity in all patients and may have introduced some degree of misclassification. Importantly, this limitation also introduces the potential for confounding by indication, whereby treatment exposure, particularly biologic therapy, may primarily reflect greater underlying disease severity rather than a direct pharmacologic effect on uveitis risk, thereby constraining our ability to isolate independent treatment effects. Accordingly, the observed association between biologic therapy and uveitis risk should be interpreted with caution. Nevertheless, classification of psoriasis severity based on treatment exposure is a commonly accepted methodological approach in large-scale epidemiological studies, including prior population-based investigations of uveitis risk, particularly when standardized clinical severity indices are not available [11,22,42,43,44,45].

Second, although both anterior and posterior uveitis diagnoses (ICD-9 codes 364. and 363., respectively) were extracted from the database, the aggregated output provided for analysis did not differentiate between anatomical subtypes. This limitation precluded stratified analyses according to uveitis phenotype and may have obscured potential differences in risk across specific anatomical forms. At the same time, while subtype-specific analyses could have provided additional clinical insight, further stratification would have substantially reduced the number of outcome events and potentially limited statistical power, thereby affecting the precision of risk estimates. Additionally, our database recorded only the initial diagnosis of uveitis, precluding any evaluation of recurrence patterns, chronicity, or risk factors associated with recurrent disease. Third, to ensure accurate estimation of incident risk, individuals with a documented history of uveitis prior to their psoriasis diagnosis were excluded. Although this approach was methodologically necessary to maintain temporal sequencing and valid hazard ratio estimation within a longitudinal framework, this exclusion, affecting approximately 50% of potential psoriasis cases (Table 2), may have led to an underestimation of the overall burden of uveitis in this population. Finally, genetic factors, particularly HLA-B27, play a well-established role in uveitis pathogenesis and are differentially distributed across populations [46]. Notably, the prevalence of HLA-B27 is lower in Middle Eastern populations compared with Northern European populations. As individual-level genetic data were not available in the current database, we were unable to directly assess the contribution of HLA-B27 to uveitis risk. This limitation should therefore be considered when extrapolating our findings to populations with different genetic backgrounds. Despite the limitations, our study possesses several substantial strengths. Considering the scarcity of large population-based psoriasis cohorts with sufficient power to evaluate uveitis risk, our investigation provides valuable insight by leveraging a comprehensive and well-validated electronic health records database with a prolonged follow-up period. Moreover, the inclusion of patients from the Israeli population, characterized by ethnic and genetic heterogeneity, improves the generalizability of our findings and reinforces their applicability to diverse global populations.

## 5. Conclusions

This large population-based cohort study demonstrated an elevated risk of incident uveitis among patients with psoriasis, with particularly high risk observed in those with severe disease and concomitant SpA. Within the psoriasis cohort, several immune-mediated comorbidities were identified as independent predictors of uveitis development. Interestingly, biologic therapy was associated with a higher risk of uveitis compared to cDMARDs, likely reflecting underlying disease severity rather than a causal treatment effect.

These findings support the hypothesis that increased systemic inflammatory activity may drive uveitis risk through shared immunopathogenic mechanisms. Clinicians should maintain heightened vigilance for uveitis in patients with psoriasis, especially in high-risk subgroups, and consider timely referral for ophthalmologic evaluation when symptoms arise. Future studies incorporating objective severity metrics and mechanistic insights are needed to clarify the effect of various anti-inflammatory therapies on uveitis risk and refine risk stratification approaches in clinical practice.

Beyond organ-specific outcomes, chronic inflammatory skin diseases such as psoriasis exert a substantial impact on patients’ quality of life and overall well-being, particularly in the presence of extracutaneous comorbidities. Recent dermatologic literature has increasingly emphasized the importance of multidisciplinary and patient-centered approaches to managing the cumulative physical and psychosocial burden of chronic skin conditions. Our findings, demonstrating a markedly elevated risk of uveitis among specific psoriasis subgroups, further underscore the clinical complexity of this population. Improved identification of patients at heightened risk for comorbidities may facilitate more personalized management strategies, potentially reducing morbidity and preserving quality of life.

## Figures and Tables

**Table 1:** Baseline characteristics of the study population.

Characteristics	Controls (*n* = 156,927)	Psoriasis (*n* = 157,360)	*p*-Value
Demographics			
Age at psoriasis onset, mean ± SD	47.7 ± 18.1	47.8 ± 18.1	0.136
Male sex, *n* (%)	78,392 (50.0%)	78,790 (50.1%)	0.519
Arab ethnicity, *n* (%)	31,020 (19.8%)	23,446 (14.9%)	<0.001
Chronic Comorbidities			
Smoking, *n* (%)	60,720 (38.7%)	69,742 (44.3%)	<0.001
Obesity, *n* (%)	43,503 (27.7%)	51,614 (32.8%)	<0.001
Diabetes Mellitus, *n* (%)	31,730 (20.2%)	36,977 (23.5%)	<0.001
Hypertension, *n* (%)	50,900 (32.4%)	56,114 (35.7%)	<0.001
Ischemic Heart Disease, *n* (%)	19,414 (12.4%)	25,027 (15.9%)	<0.001
Congestive Heart Failure, *n* (%)	6893 (4.4%)	10,676 (6.8%)	<0.001
Cerebrovascular Accident, *n* (%)	9433 (6.0%)	11,957 (7.6%)	<0.001
Peripheral Vascular Disease, *n* (%)	4182 (2.7%)	6647 (4.2%)	<0.001
Chronic Renal Failure, *n* (%)	8582 (5.5%)	12,816 (8.1%)	<0.001
Malignancy, *n* (%)	18,660 (11.9%)	24,553 (15.6%)	<0.001
Chronic Obstructive Pulmonary Disease, *n* (%)	5945 (3.8%)	9528 (6.1%)	<0.001
Asthma, *n* (%)	10,804 (6.9%)	13,674 (8.7%)	<0.001
Immune-Mediated Comorbidities			
Uveitis	1195 (0.8%)	1512 (1.0%)	<0.001
Ulcerative Colitis, *n* (%)	756 (0.5%)	1088 (0.7%)	<0.001
Crohn’s disease, *n* (%)	16 (0.2)	5 (0.1)	0.016
Systemic Lupus Erythematosus, *n* (%)	345 (0.2%)	581 (0.4%)	<0.001
Scleroderma, *n* (%)	168 (0.1%)	248 (0.2%)	<0.001
Rheumatoid Arthritis, *n* (%)	1593 (1.0%)	2877 (1.8%)	<0.001
Sarcoidosis, *n* (%)	361 (0.2%)	539 (0.3%)	<0.001
Behçet’s Disease, *n* (%)	60 (0.0%)	88 (0.1%)	0.0275
Gout, *n* (%)	2656 (1.7%)	3497 (2.2%)	<0.001

**Table 2:** Demographic and clinical characteristics of psoriasis patients with uveitis.

Characteristics	Uveitis (*n* = 1512)
Age at psoriasis, mean ± SD	53.2 ± 17.6
Age at uveitis, mean ± SD	54.3 ± 17.9
Male sex, *n* (%)	710 (47.0%)
Arab ethnicity, *n* (%)	222 (14.7%)
Time difference between onset of psoriasis and onset of uveitis (years), median (Range)	5.40 (0–21.0)
Psoriasis type	
Mild, *n* (%)	1238 (81.9)
Severe, *n* (%)	274 (18.1)
SpA related diseases	
Inflammatory Arthritis, *n* (%) *	270 (17.9%)
Inflammatory Bowel Disease, *n* (%)	79 (5.2%)
Uveitis diagnosis relative to psoriasis	
Uveitis diagnosed before psoriasis, *n* (%)	678 (44.8%)
Uveitis diagnosed after psoriasis, *n* (%)	834 (55.2%)
Treatment	
Phototherapy, *n* (%)	125 (8.3%)
Methotrexate, *n* (%)	237 (15.7%)
Anti-TNF, *n* (%)	206 (13.6%)
Anti-IL-12/23, *n* (%)	24 (1.6%)
Anti-IL-17, *n* (%)	35 (2.3%)
Anti-IL- 23, *n* (%)	11 (0.7%)
Multiple lines, *n* (%)	54 (3.6%)

* Inflammatory Arthritis: psoriatic arthritis (PsA), ankylosing spondylitis (AS).

**Table 3:** Incidence of uveitis in psoriasis patients compared to controls, time to event analysis, with subgroups analyses.

Variables	Controls (*n* = 156,434)	All Psoriasis (*n* = 156,682)	Mild Psoriasis (*n* = 131,106)	Severe Psoriasis (*n* = 9928)	Psoriasis with SpA-Related Comorbidities (*n* = 15,648)
Events, *n*	702	834	589	72	173
Follow-up time, person-years	1,985,618	1,990,558	1,631,191	135,428	223,939
Follow-up time, median (IQR)	12.6 (7.8–17.7)	12.6 (7.8–17.7)	12.2 (7.6–17.3)	14.1 (8.8–18.6)	15.2 (9.7–19.3)
Incidence rate per 1000 person-years	0.354	0.419	0.361	0.532	0.773
Unadjusted HR (95%CI)	reference	1.19 (1.07 to 1.31) *	1.02 (0.91 to 1.14)	1.52 (1.19 to 1.93) *	2.22 (1.88 to 2.62) *
Age-and-sex adjusted HR (95%CI)	reference	1.80 (1.50 to 2.15) *	1.01 (0.91 to 1.13)	1.59 (1.25 to 2.03) *	2.21 (1.87 to 2.61) *

* *p*-value < 0.001. Abbreviations: CI, confidence interval; HR, hazard ratio; IQR, interquartile-range; SpA, spondyloarthritis.

**Table 4:** Predictors of uveitis development in psoriasis, including pre-event treatment exposures.

Characteristics	Univariable OR (95% CI)	*p*-Value	Multivariable OR (95% CI)	*p*-Value
Demographics				
Age, years †	1.01 (1.01–1.01)	<0.001	1.00 (0.99–1.01)	0.8
Female sex	1.14 (1.00–1.31)	0.055	1.20 (0.99–1.47)	0.069
Arab ethnicity	1.21 (0.69–2.31)	0.5	1.08 (0.47–3.13)	0.9
Chronic comorbidities				
Obesity (BMI ≥ 30)	1.18 (0.96–1.45)	0.10	0.99 (0.80–1.23)	>0.9
SpA	2.37 (2.00–2.80)	<0.001	1.83 (1.40–2.37)	<0.001
Diabetes	1.63 (1.41–1.88)	<0.001	1.53 (1.22–1.92)	<0.001
Hypertension	1.45 (1.26–1.66)	<0.001	1.05 (0.81–1.35)	0.7
IHD	1.05 (0.70–1.50)	0.8	1.04 (0.63–1.62)	0.9
CHF	1.13 (0.86–1.44)	0.4	0.87 (0.60–1.24)	0.5
CVA	1.35 (1.07–1.68)	0.010	1.04 (0.73–1.44)	0.8
Malignancy	1.50 (1.26–1.76)	<0.001	1.21 (0.94–1.54)	0.14
SLE	2.99 (1.43–5.45)	0.001	2.46 (1.0–5.44)	0.049
Sarcoidosis	7.99 (4.98–12.1)	<0.001	7.14 (3.84–12.1)	<0.001
Treatments				
cDMARDS				
NO = 686/144,675 (0.47%)				
YES = 100/11,959 (0.84%)	1.77 (1.43–2.17)	<0.001	1.77 (1.42–2.17)	<0.001
Phototherapy				
NO = 755/145,093 (0.52%)				
YSE = 54/11,564 (0.47%)	0.90 (0.67–1.17)	0.4	0.93 (0.70–1.21)	0.6
Biologics (all)				
NO = 720/149,773 (0.48%)				
YSE = 73/6868 (1.06%)	2.22 (1.73–2.81)	<0.001	2.41 (1.87–3.05)	<0.001
Monoclonal anti-TNFα				
NO = 736/151,054 (0.49%)				
YSE = 57/5587 (1.02%)	2.11 (1.59–2.73)	<0.001	2.29 (1.72–2.98)	<0.001
Etanercept				
NO = 778/154,086 (0.50%)				
YSE = 42/2582 (1.63%)	3.26 (2.35–4.40)	<0.001	3.37 (2.42–4.54)	<0.001
Anti-IL17				
NO = 810/154,677 (0.52%)				
YSE = 7/1988 (0.35%)	0.67 (0.29–1.31)	0.3	0.72 (0.31–1.40)	0.4

† For every 1-years increment. Abbreviations: cDMARDS, conventional disease-modifying antirheumatic drug (cyclosporine, methotrexate); CI, confidence interval; OR, odds ratio; SLE, systemic lupus erythematosus; CVA, cerebrovascular accident; CHF, congestive heart failure; IHD, Ischemic Heart Disease; SpA, spondyloarthritis.

## Data Availability

The datasets presented in this article are not readily available due to the privacy policy of CHS. Requests to access the datasets should be directed to CHS.

## References

[1] Bernstein C.N., Wajda A., Blanchard J.F. (2005). The Clustering of Other Chronic Inflammatory Diseases in Inflammatory Bowel Disease: A Population-Based Study. Gastroenterology.

[2] Neimann A.L., Shin D.B., Wang X., Margolis D.J., Troxel A.B., Gelfand J.M. (2006). Prevalence of cardiovascular risk factors in patients with psoriasis. J. Am. Acad. Dermatol..

[3] Armstrong A.W., Mehta M.D., Schupp C.W., Gondo G.C., Bell S.J., Griffiths C.E.M. (2021). Psoriasis Prevalence in Adults in the United States. JAMA Dermatol..

[4] Köse B., Uzlu D., Erdöl H. (2022). Psoriasis and uveitis. Int. Ophthalmol..

[5] Bachelez H., van de Kerkhof P.C.M., Strohal R., Kubanov A., Valenzuela F., Lee J.H., Yakusevich V., Chimenti S., Papacharalambous J., Proulx J. (2015). Tofacitinib versus etanercept or placebo in moderate-to-severe chronic plaque psoriasis: A phase 3 randomised non-inferiority trial. Lancet.

[6] Smith C.H., Yiu Z.Z.N., Bale T., Burden A.D., Coates L.C., Edwards W., MacMahon E., Mahil S., McGuire A., Murphy R. (2020). British Association of Dermatologists guidelines for biologic therapy for psoriasis 2020: A rapid update. Br. J. Dermatol..

[7] Rosenbaum J.T. (2015). Uveitis in spondyloarthritis including psoriatic arthritis, ankylosing spondylitis, and inflammatory bowel disease. Clin. Rheumatol..

[8] Zeboulon N., Dougados M., Gossec L. (2008). Prevalence and characteristics of uveitis in the spondyloarthropathies: A systematic literature review. Ann. Rheum. Dis..

[9] Agrawal R., Murthy S., Sangwan V., Biswas J. (2010). Current approach in diagnosis and management of anterior uveitis. Indian J. Ophthalmol..

[10] Chen C.H., Lin K.C., Chen H.A., Liao H.T., Liang T.H., Wang H.P., Chou C.-T. (2007). Association of acute anterior uveitis with disease activity, functional ability and physical mobility in patients with ankylosing spondylitis: A cross-sectional study of Chinese patients in Taiwan. Clin. Rheumatol..

[11] Patt Y.S., Ben-Shabat N., Sharif K., David P., Patt C., Elizur Y., Shani U., Zacay G., Watad A., Amital H. (2024). Unraveling the connection: Uveitis prevalence and risk factors in psoriasis patients—A population-based study. J. Dermatol..

[12] Hijazi N., Gazitt T., Haddad A., Elias M., Kassem S., Feldhamer I., Cohen A.D., Sar S., Tomkins-Netzer O., Saliba W. (2024). The risk factors for uveitis among psoriatic arthritis patients: A population-based cohort study. Clin. Rheumatol..

[13] Kilic B., Dogan U., Parlak A.H., Goksugur N., Polat M., Serin D., Ozmen S. (2013). Ocular findings in patients with psoriasis. Int. J. Dermatol..

[14] Knox D.L. (1979). Psoriasis and intraocular inflammation. Trans. Am. Ophthalmol. Soc..

[15] Wu D., Cai R., Pang Y., Ma J., Chen B., Bao S., Zheng K., Jiang W., Qi Y., Li N. (2025). Bidirectional association between uveitis and psoriasis: A systematic review and meta-analysis. Clin. Exp. Med..

[16] Wang C.Y., Lin T.Y., Wang T.Y., Chi C.C. (2026). Ocular Comorbidities of Psoriasis: A Systematic Review and Meta-analysis of Observational Studies. Am. J. Ophthalmol..

[17] Egeberg A., Khalid U., Gislason G.H., Mallbris L., Skov L., Hansen P.R. (2015). Association of Psoriatic Disease with Uveitis. JAMA Dermatol.

[18] Patt Y.S., Ben-Shabat N., Sharif K., Patt C., Elizur Y., Arow M., Cohen A.D., Watad A., McGonagle D., Amital H. (2024). The Association Between Sarcoidosis and Malignancy: A Comprehensive Population-Based Cohort Study. J. Clin. Med..

[19] Patt Y.S., Ben-Shabat N., Fisher L., Sharif K., Arow M., Lassman S., Watad A., Skuja V., Shtewe A.H., McGonagle D. (2024). Increased risk of osteoporosis and femoral neck fractures in patients with familial Mediterranean fever-a large retrospective cohort study. Rheumatology.

[20] Omar S., Helaly H. (2018). Prevalence of ocular findings in a sample of Egyptian patients with psoriasis. Indian J. Dermatol. Venereol. Leprol..

[21] Radtke M.A., Schäfer I., Glaeske G., Jacobi A., Augustin M. (2017). Prevalence and comorbidities in adults with psoriasis compared to atopic eczema. J. Eur. Acad. Dermatol. Venereol..

[22] Chi C.C., Tung T.H., Wang J., Lin Y.S., Chen Y.F., Hsu T.K., Wang S.-H. (2017). Risk of Uveitis Among People with Psoriasis. JAMA Ophthalmol..

[23] Kim B.R., Choi S.W., Choi C.W., Lee K.H., Kim M.J., Woo S.J., Youn S.W. (2023). Risk of uveitis in patients with psoriasis in Korea: A nationwide population-based cohort study. J. Eur. Acad. Dermatol. Venereol..

[24] Ziade N.R. (2017). HLA B27 antigen in Middle Eastern and Arab countries: Systematic review of the strength of association with axial spondyloarthritis and methodological gaps. BMC Musculoskelet. Disord..

[25] Yeung I.Y.L., Popp N.A., Chan C.C. (2015). The Role of Sex in Uveitis and Ocular Inflammation. Int. Ophthalmol. Clin..

[26] Giorgiutti S., Jacquot R., El Jammal T., Bert A., Jamilloux Y., Kodjikian L., Sève P. (2023). Sarcoidosis-Related Uveitis: A Review. J. Clin. Med..

[27] Shoughy S.S., Tabbara K.F. (2016). Ocular findings in systemic lupus erythematosus. Saudi J. Ophthalmol..

[28] Jindal D.A., Alshammari N., Dihan Q., Chauhan M.Z., Gupta V., Soliman M.K., Sallam A.B. (2025). The Association of Diabetes Mellitus with Uveitis: A Real-World Data Analysis. Ocul. Immunol. Inflamm..

[29] Jiang Q., Li Z., Tao T., Duan R., Wang X., Su W. (2021). TNF-α in Uveitis: From Bench to Clinic. Front. Pharmacol..

[30] Leclercq M., Desbois A.C., Domont F., Maalouf G., Touhami S., Cacoub P., Bodaghi B., Saadoun D. (2020). Biotherapies in Uveitis. J. Clin. Med..

[31] Kakkassery V., Mergler S., Pleyer U. (2010). Anti-TNF-α Treatment: A Possible Promoter in Endogenous Uveitis? Observational Report on Six Patients: Occurrence of Uveitis Following Etanercept Treatment. Curr. Eye Res..

[32] Wendling D., Paccou J., Berthelot J.M., Flipo R.M., Guillaume-Czitrom S., Prati C., Dernis E., Direz G., Ferrazzi V., Ristori J.-M. (2011). New Onset of Uveitis During Anti-Tumor Necrosis Factor Treatment for Rheumatic Diseases. Semin. Arthritis Rheum..

[33] Egwuagu C.E., Alhakeem S.A., Mbanefo E.C. (2021). Uveitis: Molecular Pathogenesis and Emerging Therapies. Front. Immunol..

[34] Guo K., Zhang X. (2021). Cytokines that Modulate the Differentiation of Th17 Cells in Autoimmune Uveitis. J. Immunol. Res..

[35] Chen Y.H., Lightman S., Calder V.L. (2021). CD4+ T-Cell Plasticity in Non-Infectious Retinal Inflammatory Disease. Int. J. Mol. Sci..

[36] Lin W., Liu T., Wang B., Bi H. (2019). The role of ocular dendritic cells in uveitis. Immunol. Lett..

[37] Diani M., Altomare G., Reali E. (2016). T Helper Cell Subsets in Clinical Manifestations of Psoriasis. Immunol. Res..

[38] Furiati S.C., Catarino J.S., Silva M.V., Silva R.F., Estevam R.B., Teodoro R.B., Pereira S.L., Ataide M., Rodrigues V., Rodrigues D.B.R. (2019). Th1, Th17, and Treg Responses are Differently Modulated by TNF-α Inhibitors and Methotrexate in Psoriasis Patients. Sci. Rep..

[39] Rendon A., Schäkel K. (2019). Psoriasis Pathogenesis and Treatment. Int. J. Mol. Sci..

[40] Ooi K.G.J., Galatowicz G., Calder V.L., Lightman S.L. (2006). Cytokines and Chemokines in Uveitis—Is there a Correlation with Clinical Phenotype?. Clin. Med. Res..

[41] El-Asrar A.M.A., Struyf S., Kangave D., Al-Obeidan S.S., Opdenakker G., Geboes K., Van Damme J. (2011). Cytokine profiles in aqueous humor of patients with different clinical entities of endogenous uveitis. Clin. Immunol..

[42] Yang Y.W., Keller J.J., Lin H.C. (2011). Medical comorbidity associated with psoriasis in adults: A population-based study. Br. J. Dermatol..

[43] Wan J., Wang S., Haynes K., Denburg M.R., Shin D.B., Gelfand J.M. (2013). Risk of moderate to advanced kidney disease in patients with psoriasis: Population based cohort study. BMJ.

[44] Chang Y., Chen T., Liu P., Chen Y., Chen Y., Huang Y., Jih J.S., Chen C.C., Lee D.D., Wang W.J. (2009). Epidemiological Study of Psoriasis in the National Health Insurance Database in Taiwan. Acta Derm. Venereol..

[45] Tsai T.F., Wang T.S., Hung S.T., Tsai P.I.C., Schenkel B., Zhang M., Tang C.-H. (2011). Epidemiology and comorbidities of psoriasis patients in a national database in Taiwan. J. Dermatol. Sci..

[46] Durrani K., Foster C.S. (2005). Psoriatic uveitis: A distinct clinical entity?. Am. J. Ophthalmol..

